# Association of Exposure to Particular Matter and Carotid Intima-Media Thickness: A Systematic Review and Meta-Analysis

**DOI:** 10.3390/ijerph121012924

**Published:** 2015-10-15

**Authors:** Xiaole Liu, Hui Lian, Yanping Ruan, Ruijuan Liang, Xiaoyi Zhao, Michael Routledge, Zhongjie Fan

**Affiliations:** 1 Department of Cardiology, Peking Union Medical College Hospital, Peking Union Medical College, Chinese Academy of Medical Sciences, Beijing 100730, China; E-Mails: lxlryyc@sina.com (X.L.); lianhui1988@163.com (H.L.); yanping.ruan@163.com (Y.R.); 18911122174@163.com (R.L.); zhaoxy0909@sina.com (X.Z.); 2 Leeds Institute of Cardiovascular & Metabolic Medicine, University of Leeds, Leeds LS2 9JT, UK; E-Mail: medmnr@leeds.ac.uk

**Keywords:** air pollution, PM_2.5_, PM_10_, carotid intima-media thickness, subclinical atherosclerosis, meta-analysis

## Abstract

*Background*: Long time exposure to particular matter has been linked to myocardial infarction, stroke and blood pressure, but its association with atherosclerosis is not clear. This meta-analysis was aimed at assessing whether PM_2.5_ and PM_10_ have an effect on subclinical atherosclerosis measured by carotid intima-media thickness (CIMT). *Methods*: Pubmed, Ovid Medline, Embase and NICK between 1948 and 31 March 2015 were searched by combining the keywords about exposure to the outcome related words. The random-effects model was applied in computing the change of CIMT and their corresponding 95% confidence interval (95% CI). The effect of potential confounding factors was assessed by stratified analysis and the impact of traffic proximity was also estimated. *Results*: Among 56 identified studies, 11 articles satisfied the inclusion criteria. In overall analysis increments of 10 μg/m^3^ in PM_2.5_ and PM_10_ were associated with an increase of CIMT (16.79 μm; 95% CI, 4.95–28.63 μm and 4.13 μm; 95% CI, −5.79–14.04 μm, respectively). Results shown in subgroup analysis had reference value for comparing with those of the overall analysis. The impact of traffic proximity on CIMT was uncertain. *Conclusions*: Exposure to PM_2.5_ had a significant association with CIMT and for women the effect may be more obvious.

## 1. Introduction

The association between air pollution (especially particular matter) and cardiovascular disease (CVD) or their risk factors has been demonstrated by a great number of epidemiological and experimental studies [1,2,3,4,5,6]. Among the size fractions of particular matter, long term exposure to ambient and individual particular matter less than 2.5 μm in diameter (PM_2.5_) is responsible for morbidity and mortality of cardiovascular events [7].

Atherosclerosis is a chronic process and mainly affects the aorta, coronary artery and cerebral artery, which often leads to serious consequences like lumen occlusion and plaque rupture. It is the major pathological process of heart disease and stroke. In Western developed countries, atherosclerosis account for about 50% of all deaths [8,9,10]. Epidemiological studies have suggested that the degree of atherosclerosis can be measured by CIMT to forecast population’s future cardiovascular risk [11,12,13].

Several studies of long term exposure to particular matter (PM_2.5_, PM_10_) have shown that the higher particular matter concentrations were associated with increased CIMT. However, not all studies had found significant results, so the association between particular matter and CIMT is still uncertain. We therefore systematically reviewed the studies examining the effect of particular matter (PM_2.5_, PM_10_) on CIMT to quantify this effect.

## 2. Materials and Methods

### 2.1. Search Strategy and Eligibility Criteria

We performed a comprehensive databases search in PubMed, Ovid Medline, Embase and CNKI using the following key words: “air pollution”, “air pollutants”, “particular matter”, “PM_2.5_”, “PM_10_”, “meteorological factor”, “carotid intima-media thickness”, “Carotid atherosclerosis”, “carotid IMT (intima-media thickness)”, “CIMT” and “subclinical atherosclerosis”. The publication date of literature was limited between 1948 and 31 March 2015.Literatures were included if they were population-based studies, which not only reported the association between particular matter(PM_2.5_ or PM_10_) and CIMT, but also provided original data for particular matter(PM_2.5_ or PM_10_) and carotid artery intima-media thickness. There were no language restrictions. We excluded duplicates, summaries, reviews, letters, commentaries and editorials, toxicological studies, case reports and case series. In this way we selected 13 studies meeting the inclusion criteria. However, two of these studies, which only presented the median value or the percentage change of CIMT, didn’t have adequate data for CIMT. We contacted authors for detail data and no answer was obtained, so the two studies were excluded and 11 studies were finally included in our meta-analyses.

### 2.2. Study Selection

By screening all titles and abstracts potentially eligible studies were selected by two independent investigators (Xiaole Liu and Hui Lian). Then the eligibility of the study for the meta-analysis was picked out by reading the full text of the potentially eligible studies. If the two reviewers had disagreements, a third reviewer (Ruijuan Liang) would help adjudicate conflicts. 

### 2.3. Data Extraction

On the basis of in-depth reading of all eligible literatures we extracted the useful data and enter it in an advance designed standardized information table, which presented a comprehensive description of the study characteristics, including title, first author, journal publication year, study design, location and period, characteristics of the participants (age, sex, physical condition, sample size), measurement of CIMT, data analysis model, exposure measurement, effect measurement and confounding factors adjusted (age, sex, race, education, temperature, humidity and so on). Two reviewers (Xiaole Liu and Hui Lian) completed the operation of data extraction respectively and then compared. In case of conflict, a third reviewer (Ruijuan Liang) was asked to judge and make a decision.

### 2.4. Statistical Analysis

We transformed the value of CIMT with 95% CIs from each study for a standardized increment per 10 μg/m^3^ in particular matter (PM_2.5_, PM_10_) and took it as our outcome. In addition, we hypothesizeda linear relation between exposure and outcome because most studies used linear regression models. On account of different study designs, geographical settings, methods of exposure and CIMT measurements, characteristics of participants and exposure durations, heterogeneity existed in the studies. Therefore, we used the random-effects model, which accounts for both within and between studies heterogeneity to pool the summary-effect estimates. According to the weight (1/SE^2^) of each study account for the total, we calculated the overall effect. Standard I^2^ statistic was applied to tests for heterogeneity in order to quantify inconsistencies between studies.I^2^ values of 25% or less, 50% and 75% or more stand for low, moderate and high heterogeneity respectively.

We also did subgroup analyses stratifying studies performed by study design (cross-sectional *vs.* longitudinal), sex (female *vs.* male), education (low education *vs.* high education), treatment (lipid-lowering treatment *vs.* no lipid-lowering treatment). In addition, association between traffic proximity and CIMT was also assessed. To estimate the potential publication bias, funnel plots were constructed and we also tested them using the Egger regression test due to the limitations of funnel plot. Statistical analyses were conducted using Stata software (Stata Corp., College Station, TX, USA). Statistical significance was taken as two-sided *p *< 0.05 with the exception of the heterogeneity assessment, which was considered statistically significant at *p *< 0.01.

## 3. Results

### 3.1. Literature Search

**Figure 1:**
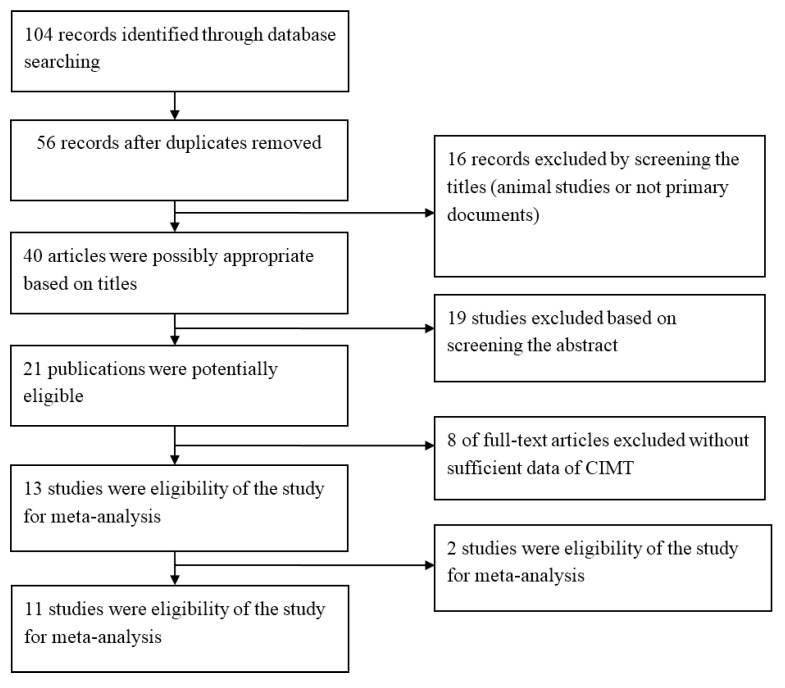
Flow chart of studies selection in the meta-analysis.

104 initial records were retrieved by searching databases and 56 remained after removing duplicates. Then by screening the titles and abstracts, 35 articles were excluded, which were animal studies or not primary documents (review, letter). After that we read the full text of the remaining 21 studies and determined 13 of them fulfilled the eligibility criteria. Whereas two studies didn’t present sufficient data for mean value of CIMT and their authors didn’t respond to our e-mails requesting information [14,15], 11 articles were finally eligibility of the review and meta-analysis. Figure 1 show a flow of information through the different study screening phases in our meta-analysis.

### 3.2. Study Characteristics

The characteristics of included studies are presented in the Table 1. Among the eligible studies, nine used cross-sectional design, one used longitudinal design, and another one used both study designs. Study locations were mainly USA (six studies), three studies were from Europe (one of them including four cohorts), one study was from Canada and one was from Taiwan. The sample size of participants ranged from 509 to 6256. The populations of these studies were all adults and most of them were healthy. Four studies selected the population from a same existing cohort, participants of which aged from 45 to 84 and without preexisting clinically apparent cardiovascular disease. In addition, one study was conducted in young adults aged from 18 to 27.

**Table 1:** Characteristics of included studies.

First Author,Year [Reference No.]	Location	Period	Study design	Samplesize	Age (Years)	Exposure Measurement	Statistical Analysis
Su, 2015 [16]	Taiwan	2009–2011	cross-sectional	689	35–65	Individual	multiple linearregression model
Perez, 2015 [17]	Europe	1997–2009	cross-sectional	9183	42–68	Individual	linear regression model
Kim, 2014 [18]	USA	2000–2002	cross-sectional	5488	45–84	individual	multiple linearregression model
Gan, 2014 [19]	Canada	2004–2011	longitudinal	509	30–65	individual	general linear regression model
Sun, 2013 [20]	USA	2000–2002	cross-sectional	6256	45–84	ambient	multiple linearregression model
Adar, 2013 [21]	USA	2000–2005	cross-sectional	5660	45–84	individual	longitudinal mixed model
Breton, 2012 [22]	USA	2007–2009	cross-sectional	768	18–27	ambient	linear regression model
Tonne, 2012 [23]	Britain	2002–2006	cross-sectional	2348	55–67	individual	generalized linear regression models
Künali, 2010 [24]	USA	1994–2006	cross-sectional, longitudinal	1483	>30	ambient	linear regression model
Lenters, 2010 [25]	The Netherlands	1999–2000	cross-sectional	745	45–84	individual	multiple linearregression model
Künali, 2005 [26]	USA	1998–2003	cross-sectional	798	>40	ambient	linear regression model

Particular matter in 10 studies was PM_2.5_, in one study it was PM_10_ and in three studies both. The concentrations of PM_10_ and PM_2.5_ were estimated in ambient or individual-levels. Linear regression model was used to evaluate the association between air pollution and CIMT in most studied. The number of potential confounding factors included in the studies varied, most adjusted for age, sex, race/ethnicity, body mass index (BMI), smoking status, education, and low-density lipoprotein cholesterol (LDL-C) in the results.

### 3.3. Analysis

**Figure 2:**
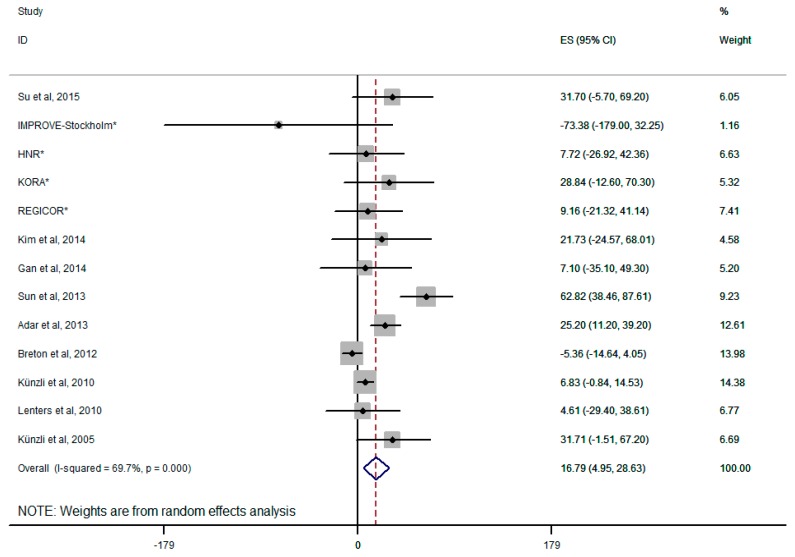
Forest plot for overall analysis of the association between PM_2.5_ and CIMT (random-effects model); * four on-going European cohort analyzed by Perez *et al. *[17].

The results from the random-effects meta-analysis of the relationship between exposure to PM_2.5_ and CIMT are shown in Figure 2. When the concentration of PM_2.5_ increased 10 μg/m^3^ in the evaluation of overall combination, its relationship with CIMT reached statistical significance (the increment of CIMT is 16.79 μm; 95% CI, 4.95–28.63 μm). The heterogeneity observed for 10 studies was small-medium (*I^2^* = 67.6%). When some of the eligible studies were pooled with an additional adjustment for education and income, the summary estimate was attenuated to 16.68 μm (95% CI, 4.93–28.43 μm), results were not shown in the table.

**Figure 3:**
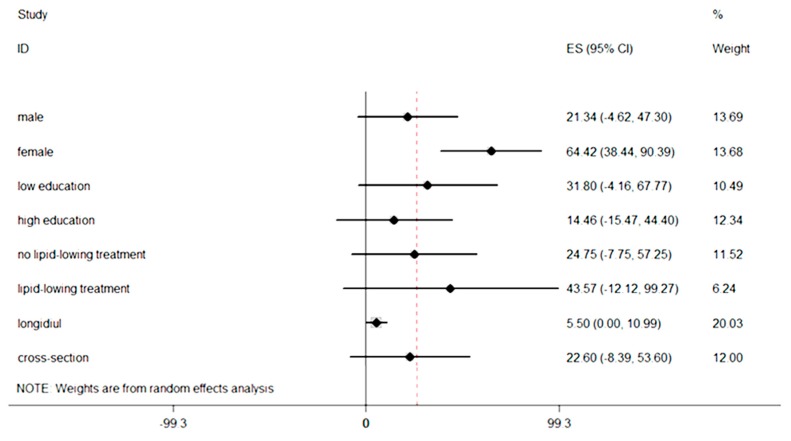
Subgroup analyses based on sex, education, treatment and study design.

There was no statistical evidence of publication bias in overall analyses. In Egger’s test we got *p *= 0.211. The subgroup analysis about PM_2.5_ and CIMT was conducted by sex (Adar *et al.* [21]; Lenters *et al. *[25]; Künzli *et al. *[26]), education (Adar *et al.* [21]; Künzli *et al. *[24]; Lenters *et al. *[25]), lipid-lowering treatment (Adar *et al.* [21]; Künzli *et al.* [24]; Künzli *et al.* [26]) and study design. Significant association between PM_2.5_ and CIMT was found with female, the summary estimate of which was larger than overall analysis (64.42 μm; 95% CI, 38.44–90.39 μm). However, the association with male had no statistic significant. Similarly, in longitudinal study design a weak correlation between exposure to PM_2.5_ and CIMT was represented (5.50 μm; 95% CI, 0.00–10.99 μm), comparing with no significant association in cross-sectional study design (22.60 μm; 95% CI, −8.39–53.60 μm). The subgroup analysis stratified by educational qualifications showed that people with low education had a larger effect than high education, whereas neither of them had statistic significant. The pooled estimates of low education and high education were 31.80 μm (95% CI, −4.16–67.77 μm) and 14.46 μm (95% CI,−15.47–44.40 μm), respectively. When compared the effects based on whether receive the lipid-lowering treatment, we also found a difference. Participants with a lipid-lowering treatment showed an increase of CIMT with 43.57 μm (95% CI, −12.12–99.27 μm), whereas people had no lipid-lowering treatment was 24.74 μm (95% CI,−7.75–57.25 μm). (Figure 3).

**Figure 4:**
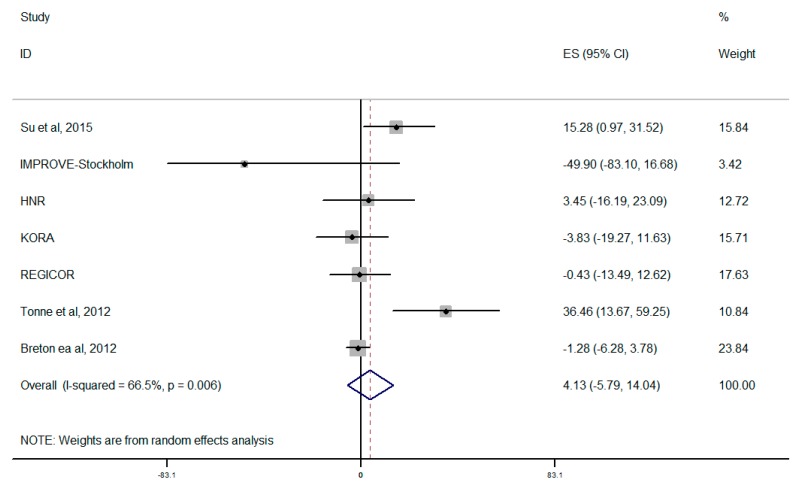
Forest plot for overall analysis of the association between PM_10_ and CIMT (random-effects model); * four on-going European cohorts analyzed by Perez *et al. *[17].

Summary estimate for PM_10_ is shown in Figure 4. When the concentration of PM_10_ increased 10 μg/m^3^ in the evaluation of overall combination, the CIMT increment is 4.13 μm (95% CI, −5.79–14.04 μm), which was inverse in comparison with PM_2.5_ but not statistically significant, though there was significant heterogeneity across the studies (*I*^2^ = 66.5% or *p *= 0.006) in associations with PM_10_.

## 4. Discussion

This study is, to our knowledge, the first meta-analysis which estimates the effects of exposure to particular matter (PM_2.5_, PM_10_) on CIMT, an accepted measure of the progression of atherosclerosis [27,28]. In overall estimation we observed a significant and positive association between PM_2.5_ and CIMT. When compared with the overall analysis, subgroup analyses were associated with lower heterogeneity and had the same direction of estimated effect. Therefore, the association was robust.

Two studies we excluded due a lack of sufficient data for mean value of CIMT showed similar results. One is in Germany [14]: Median CIMT of the 3380 analyzed participants was 0.66 mm (interquartile range 0.16 mm). An interdecile range increase in PM_2.5_ (4.2 μg/m^3^), PM_10_ (6.7 μg/m^3^), and distance to high traffic (1939 m) were associated with a 4.3% (95% CI, 1.9%–6.7%), 1.7% (95% CI, −0.7%–4.1%), and 1.2% (95% CI,−0.2%–2.6%) increase in CIMT, respectively; The other one is in the USA [15]: Intimal-medial thickness was weakly, positively associated with exposures to particulate matter <10 μmin aerodynamic diameter and <2.5 μm in aerodynamic diameter after controlling for age, sex, race/ethnicity, socioeconomic factors, diet, smoking, physical activity, blood lipids, diabetes, hypertension, and body mass index (1%–4% increase per 21-μg/m^3^ increase in particulate matter <10 μm in aerodynamic diameter or a 12.5-μg/m^3^ increase in particulate matter <2.5 μm in aerodynamic diameter). Results were consistent from a qualitative angle and didn’t affect the results in our paper.

CIMT results from the processes of cumulative atherogenesis and is a predictor of cardiovascular events. In comparison with pulse wave velocity and augmentation index, which are affected by changes in blood pressure, CIMT is quite easy to be measured due to little short-term variation. Therefore, previous environment studies applied it to measure the degree of atherosclerosis [27,29,30,31]. Inflammatory dysfunction [32], the excitation of oxidative stress and autonomic imbalance are thought to be the potential pathways, by which particular matter is associated with atherogenesis. Then these potential pathways lead to endothelial dysfunction and reduction of vascular reactivity, which have been proved to be early manifestations of atherosclerosis by many studies. Endothelial dysfunction, as a possible and important mechanism, is an initial step in atherosclerosis. Several studies have pointed out a relationship between endothelial function and particulate air pollution [33,34,35]. Santoro* et al.* [36] demonstrated that endothelial dysfunction can occur without structural atherosclerotic changes in young women with endometriosis, which underlined the importance to investigate this parameter especially in young and healthy people, to confirm the precocity of endothelial dysfunction respect to intima-media thickness. Hoffmann* et al. * [37]showed fine particulate matter exposure was associated with coronary artery calcification (CAC), which is related in relevant ways to CIMT. Wilker* et al. *[38] reported annual mean black carbon concentration was associated with CIMT in the elderly. These findings supported an association between long-term air pollution exposure and atherosclerosis. Many animal experiments also supported the viewpoint that exposure to PM_2.5_ may contribute to the process of atherosclerosis by potential mechanisms including bone marrow stimulation, release of monocytes and altered vasomotor tone [39,40,41].Exposure of apolipoproteinE mice to PM_2.5_ had an effect on altered vasomotor tone, vascular inflammation and increase of aortic atherosclerosis [39]. In addition, animal experiments found other biologic mechanisms, which connect particulate matter exposure with the progress of atherosclerosis in a long-term period, that exposure to particular matter may influence blood pressure, autonomic function and low density lipoprotein oxidation [42,43,44,45,46].

As shown in Figure 3, we found the estimate effect was larger in women. The result suggested that gender may influence the association between PM_2.5_ and CIMT. One potential mechanism is the secretion of androgen. In addition, compared with young men, elderly men with less androgen have higher risk for atherosclerosis [26].The other one was that females had slightly greater airway reactivity than males. Thus, compared with males, we might find dose-response relations more easily in females [47]. Although previous studies also reported that particular matter had greater effect estimates in women [47,48], the effect modification by gender remained unclear and further investigations are needed [49]. Although we didn’t discuss the effects of age due to limited number of included studies, previous literature showed a linkage among sex, age and atherosclerosis [50]. If more research were conducted in the future, we can perform a subgroup analysis by age.

Participants with lower education were more vulnerable when exposed to PM_2.5_. One of the reasons was that because of poor living conditions, people with lower education are more likely to live near busy roads and expose to multiple air pollutants. For example, higher concentrations of some air pollutants have been demonstrated among disadvantaged groups [51]. Studies for Scandinavian indicated the discrepancy of personal exposures to particular matter among people with different education and occupation [52,53]. Another reason was that a majority of people receiving lower education have poor living conditions and don’t have ability to get enough nutrition, such as antioxidant polyunsaturated fatty acids and vitamins, which may protect against adverse effects of particular matter [54]. Furthermore, people with lower socioeconomic status (SES) have a higher prevalence of preexisting diseases and usually receive inferior medical treatment for them, which leads to higher sensitivity of air pollution-related health hazards.

Our study found people with lipid-lowering treatment was not significant compared with those without treatment. Some studies reported that participants taking lipid-lowering medications at baseline represented stronger association between CIMT and PM_2.5_ [24,26]. Hyperlipidemic rabbits experiments had demonstrated this conclusion [40,55]. However, the modification direction reported in other study was inconsistent. In order to illuminate the relevance of lipid and statin status, future researches can be conducted among cohorts with familial hypercholesteremia [56,57].

We also conducted meta-analyses among different study designs. Longitudinal studies compared with cross-sectional ones. When excluding cross-sectional studies, the relevance between PM_2.5_ and CIMT had no statistical significance because in cross-sectional studies, the study object was the whole population, which stopped the researchers from exploring person-level factors, while longitudinal studies took the individuals as study objects, so intra-individual variability could be taken into consideration. If more cohorts are built in the future, more longitudinal studies could be conducted.

Four studies [17,19,24,25] referred to the relationship between CIMT and traffic proximity. Due to different data expression, we cannot conduct meta-analysis. Living close to major roads may indicate high exposure to traffic-related exhaust emissions, such as ultrafine particles and other highly redox-active pollution especially diesel particles [58]. Perez* et al. *[17] presented that living in proximity to high traffic might also positively associated with CIMT. Künzli* et al. *[24] showed that a 10 μg/m^3^ increasing in PM_2.5_ annual was associated with 2.5 μm (95% CI, −0.3–5.4 μm) increase in CIMT, which had no significant. When living within 100 m traffic, annual CIMT increased 5.5 μm (95% CI, 0.13–10.79 μm) compared with people living away from traffic. However, living within a highway (100 m) or within a major road (50 m) was related with a non-significant 1.6 μm (95% CI, −0.15–3.42 μm) augment in CIMT per year. Lenters* et al. *[25] didn’t find consistent direction of association between traffic indicators (traffic proximity, traffic density) and CIMT. Similarly results were also represented by Gan* et al. *[19]. Several other recent studies observing the associations of CIMT with biomass fuel [59] and traffic-related air pollution [60] showed statistic significant results. Rivera* et al.* [60] reported that exposure contrasts between the 5th and 95th percentiles for NO_2_ (25 µg/m^3^), traffic intensity in the nearest street (15,000 vehicles/day), and traffic load within 100 m (7,200,000 vehicle-m/day) were related with 0.56% (95% CI, −1.5%–2.6%), 2.32% (95% CI, 0.48%–4.17%), and 1.91% (95% CI, −0.24–4.06) percent difference in IMT, respectively. Armijos* et al. *[61] examined the association of long-term exposure to traffic with CIMT in children. The results showed that children residing <100 meters from the nearest heavily trafficked road had mean and maximum CIMT increment of 15% and 11% compared to those living ≥200 m away (*p *= 0.0001). From the above, future researches should focus on the effects of traffic proximity on atherosclerosis. Furthermore, studies can also be conducted to find if CIMT is a necessary ideal marker to reflect adverse effects of atherosclerosis related with particular matter.

The results for PM_10_ presented by Perez* et al. *[17] were different from other ones. Bauer *et al. *[14] reported a 5% change (95% CI, 1.9%–8.3%) for an IQR increase of 5.2 μg/m^3^ PM_10_. While Tonne *et al. *[23] showed an insignificant association (1.8% change (95% CI, 0.6%–4.3%) per 6.7 μg/m^3^increase of PM_10_).

Diet of population can also affect the atherosclerotic development and lead to different results. Bassett *et al.* [62] indicated Trans-fatty acids in the diet are closely related to atherosclerosis measured by CIMT.

Several potential limitations should be noted in our study. Firstly, heterogeneity due to distinction between individuals and between studies was found across all researches, such as publication year, location, study design, study period, characteristics of participants, PM_2.5 _ and PM_10_ measurement, sample size, covariates in each study and measuring method of CIMT. According to existing detailed protocols about measurement technique and analysis, the CIMT was assessed noninvasively by B-mode ultra-sound imaging coupled with automatic data processing systems. However, the optimal site and used value are still uncertain [29]. Some studies utilized the value of mean CIMT of the right far common carotid wall. Other studies regarded the average of the largest IMT in the left and right carotid arteries as a person’s CIMT. Still one study used mean of all available maximum wall thicknesses. In addition, choosing to use the cross-sectional CIMT or progression of CIMT also make some differences. Health status of participants and medication use were others potential factors having effects on outcome. Different statistical methods including linear regression models and longitudinal mixed models as well as the varying adjusted factors used in different studies may also product heterogeneity. Besides the heterogeneity factors we referred above, the limited number of the included studies and inferences in our meta-analysis make it difficult for us to find other potential factors by further analysis. As a result of which, heterogeneity, publication bias and quality reduction for studies arose. Secondly, since few involved studies used a “multi-pollution” model, we only put a “single-pollution” model into use in our study, regardless of latent interactions between pollutants. Therefore, we can’t estimate the interaction of multi-pollution associated with CIMT. Thirdly, some potential confounding factors were shown by subgroup analysis and additional overall analysis, which assessed the pooled-effect after putting an additional adjustment for education and income in some included studies. Nonetheless, more confounding factors are not able to be represented by more stratified analysis due to the less number of studies and limited information they provide.

Our study provides further evidence that particular matter exposure may increase the risks of arteriosclerosis as well as the morbidity and mortality of cardiovascular diseases. Almost all of studies included in our meta-analysis used a linear regression model to estimate the relationship between particular matter (PM_2.5_, PM_10_) and CIMT. If the association is linear, it is of great value to reduce the concentration of PM_2.5_ at all levels and can provide a basis for the government to issue regulations for decreasing particular matter emissions. In view of the limited number of existing studies about association between particular matter and CIMT, more analogous studies should be conducted in the future to further verify the results. In addition, future research can also focus on distinguishing potential confounding factors that impact the effect of PM_2.5_ and PM_10_ on CIMT, such as noise, postmenopausal women, diabetics or physical exercise.

## 5. Conclusions

Our results showed that an increase in PM_2.5_ had a significant association with CIMT, which is a marker for subclinical atherosclerosis. In females the effect may be more obvious and statistically significant, while the relationship of the effect with education level and lipid-lowering treatment status is still unclear.
